# Melatonin Inhibits Androgen Receptor Splice Variant-7 (AR-V7)-Induced Nuclear Factor-Kappa B (NF-κB) Activation and NF-κB Activator-Induced AR-V7 Expression in Prostate Cancer Cells: Potential Implications for the Use of Melatonin in Castration-Resistant Prostate Cancer (CRPC) Therapy

**DOI:** 10.3390/ijms18061130

**Published:** 2017-05-31

**Authors:** Vincent Wing Sun Liu, Wing Lung Yau, Chun Wai Tam, Kwok-Ming Yao, Stephen Yuen Wing Shiu

**Affiliations:** 1 School of Biomedical Sciences, The University of Hong Kong, Hong Kong, China; vwsliu@hku.hk (V.W.S.L.); kmyao@hku.hk (K.-M.Y.); 2 Division of Nursing and Health Studies, School of Science and Technology, Open University of Hong Kong, Hong Kong, China; syau@ouhk.edu.hk (W.L.Y.); cwtam@ouhk.edu.hk (C.W.T.)

**Keywords:** melatonin, androgen receptor splice variant-7, nuclear factor-kappa B, castration-resistant prostate cancer

## Abstract

A major current challenge in the treatment of advanced prostate cancer, which can be initially controlled by medical or surgical castration, is the development of effective, safe, and affordable therapies against progression of the disease to the stage of castration resistance. Here, we showed that in LNCaP and 22Rv1 prostate cancer cells transiently overexpressing androgen receptor splice variant-7 (AR-V7), nuclear factor-kappa B (NF-κB) was activated and could result in up-regulated interleukin (*IL*)*-6* gene expression, indicating a positive interaction between AR-V7 expression and activated NF-κB/IL-6 signaling in castration-resistant prostate cancer (CRPC) pathogenesis. Importantly, both AR-V7-induced NF-κB activation and *IL-6* gene transcription in LNCaP and 22Rv1 cells could be inhibited by melatonin. Furthermore, stimulation of *AR-V7* mRNA expression in LNCaP cells by betulinic acid, a pharmacological NF-κB activator, was reduced by melatonin treatment. Our data support the presence of bi-directional positive interactions between AR-V7 expression and NF-κB activation in CRPC pathogenesis. Of note, melatonin, by inhibiting NF-κB activation via the previously-reported MT_1_ receptor-mediated antiproliferative pathway, can disrupt these bi-directional positive interactions between AR-V7 and NF-κB and thereby delay the development of castration resistance in advanced prostate cancer. Apparently, this therapeutic potential of melatonin in advanced prostate cancer/CRPC management is worth translation in the clinic via combined androgen depletion and melatonin repletion.

## 1. Introduction

Worldwide, prostate cancer is the fourth most common human cancer and the second most common cancer in men [1]. Despite the detection of most clinical cases at an early localized stage with prostate-specific antigen (PSA) screening, some patients still present late with more extensive disease. Since androgen is recognized to be the most important growth-promoting hormone in prostate cancer initiation and progression, androgen depletion therapy (ADT) in the form of medical or surgical castration to down-regulate androgen receptor (AR) signaling, is the recommended hormonal therapy for advanced or metastatic prostate cancer palliation [2]. Given that patients receiving ADT will enter an initial variable period of prostate cancer regression, and that the disease will relapse in the majority of patients with castration-resistant prostate cancer (CRPC) development, there is a significant demand for novel, effective, safe, and affordable therapies.

In the search for novel CRPC therapy, AR-mediated functions were noted to be incompletely abrogated by ADT. While prostate cancer cells may evolve mechanisms to bypass AR signaling, the growth of CRPC has in the majority of cases been shown to be dependent on sustained AR signaling despite a castration level of testosterone. Importantly, advances in the understanding of the mechanisms involved in CRPC pathogenesis, in particular aberrant AR signaling, have led to the recent successful development of new AR-targeting therapies, such as abiraterone and enzalutamide, to dampen sustained AR signaling in CRPC [3,4,5,6]. Of note, not all patients responded to abiraterone or enzalutamide in clinical trials, and some initial responders eventually developed progression of their disease [2,3,4,5,6]. Recently, androgen receptor splice variants (AR-Vs), which retain the N-terminal transactivation domain and DNA binding domains, but lack the C-terminal ligand binding domain (LBD) of AR [7,8,9,10], have been shown to be an important contributing factor for resistance to abiraterone and enzalutamide. Among the different variants, AR-V7 is the most studied member; its transcript abundance is high, and it is the only variant of which the encoded endogenous protein was demonstrated [10,11]. AR-V7 is overexpressed in CRPC compared with benign prostate or hormone-naïve prostate cancer tissue, and its expression has been associated with poor clinical prognosis [7,8,12]. Whereas AR-V7 induces castration resistance in androgen-dependent prostate cancer cell lines, knockdown of AR-V7 in CRPC cellular and xenograft models induces inhibition of growth [7]. Given that AR-V7 expression increases in CRPC xenografts treated with enzalutamide or abiraterone [13,14] and that detection of AR-V7 in circulating tumor cells from patients with CRPC may be associated with resistance to enzalutamide or abiraterone [15], constitutively active AR-V7 and related AR-Vs are likely to play a dominant role in inducing resistance to abiraterone and enzalutamide.

Instead of focusing on AR and AR-Vs, a different research approach has been adopted by our laboratory in our search for novel CRPC therapy. We target the signaling pathway of melatonin, a pineal gland neurohormone, for prostate cancer growth modulation. Initially, we demonstrated G protein-coupled melatonin MT_1_ receptor expression in human prostate cancer tissues, and showed the association of MT_1_ receptor expression with the antiproliferative action of melatonin in cellular and animal models of prostate cancer [16,17,18]. In a subsequent proof-of-concept translational clinical study, administration of melatonin to a castrated prostate cancer patient, whose prostate tumor tissue predominantly expressed MT_1_ receptors, was found to slow the progression of the patient’s relapsed CRPC [19]. Further laboratory studies enabled us to define the mechanisms underlying melatonin-induced antiproliferation in advanced prostate cancer. These involve the dual actions of MT_1_ receptor-mediated down-regulation of dihydrotestosterone (DHT)-activated AR signaling, and inhibition of activated nuclear factor-kappa B (NF-κB) signaling in prostate cancer cells [20,21,22,23]. Based on our data, we have recently proposed a novel combination therapeutic strategy of androgen depletion and melatonin repletion for the treatment of advanced or metastatic prostate cancer [22,23]. Since activation of NF-κB [24,25] and reactivation of AR signaling [26,27] are involved in prostate cancer progression and CRPC development, we reason that androgen depletion and melatonin repletion will help to prolong the progression-free survival of these patients by delaying CRPC development. To gain further insights on how to fully exploit the therapeutic potential of melatonin in advanced prostate cancer and CRPC management, the present study was conducted to investigate any functional interactions among AR-V7, NF-κB, and melatonin in human prostate cancer cells.

## 2. Results

### 2.1. Visualization of Overexpressed Enhanced Green Fluorescent Protein (EGFP)-Tagged AR and AR-V7 in Prostate Cancer Cells

**Figure 1:**
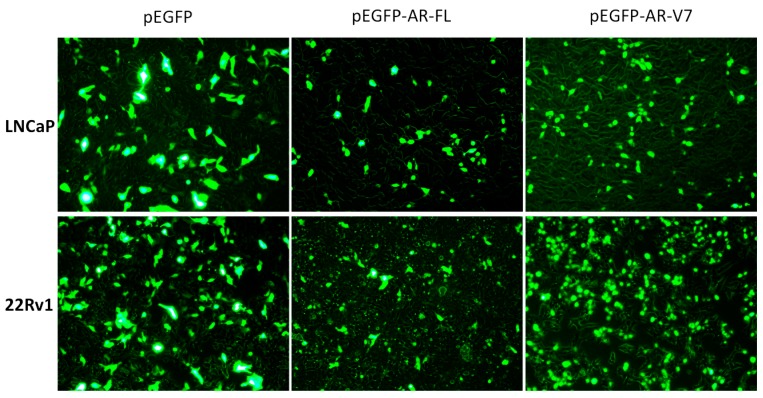
Fluorescence microscopy of LNCaP and 22Rv1 cells transfected with empty plasmid vector (pEGFP), plasmid encoding full-length androgen receptor (pEGFP-AR-FL), or plasmid encoding androgen receptor splice variant-7 (pEGFP-AR-V7). The signals were observed under a fluorescence microscope at 20× magnification. EGFP = enhanced green fluorescent protein.

Figure 1 shows the fluorescence microscopic pictures of LNCaP and 22Rv1 cells transfected with empty plasmid vector (pEGFP), plasmid encoding full-length AR (pEGFP-AR-FL), or plasmid encoding AR-V7 (pEGFP-AR-V7). EGFP was observed in both the cytoplasm and nucleus of individual prostate cancer cells transfected with the empty vector. While EGFP-AR-FL was found mainly in the nucleus and with a relatively low level of expression in the cytoplasm of individual prostate cancer cells transfected with pEGFP-AR-FL, expression of EGFP-AR-V7 was found predominantly in the nucleus of individual prostate cancer cells transfected with pEGFP-AR-V7.

### 2.2. Expression of AR and Its Variants (AR-Vs) in Prostate Cancer Cells

**Figure 2:**
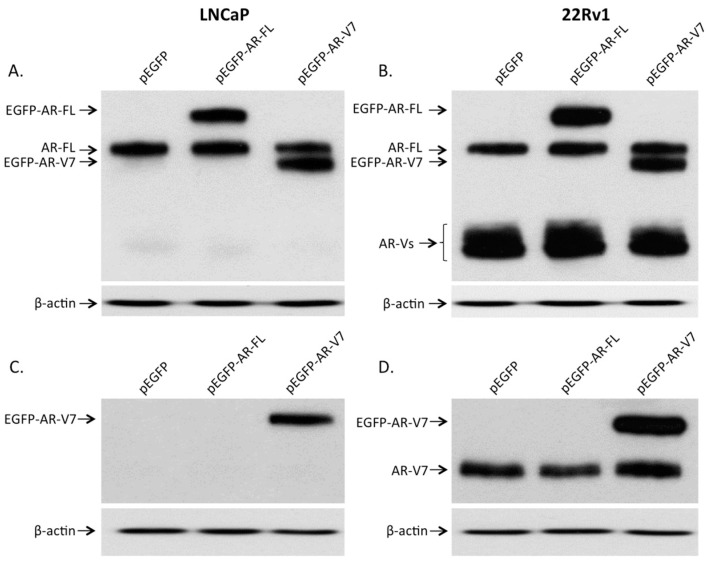
Expression of full-length androgen receptor (AR) and its variants in transfected prostate cancer cells. LNCaP (**A**) and 22Rv1 (**B**) cells were transfected with pEGFP, pEGFP-AR-FL, or pEGFP-AR-V7 expression plasmids. Immunoblot was carried out to detect the expression of endogenous and recombinant AR and its variants. Antibody against an internal region of AR was used to detect the expression of full-length AR (AR-FL) and its variants (AR-Vs). Cells transfected with pEGFP-AR-FL or pEGFP-AR-V7 expression plasmids expressed EGFP-tagged AR-FL (EGFP-AR-FL) or EGFP-tagged AR-V7 (EGFP-AR-V7), respectively. An antibody specifically against AR-V7 was also used to detect endogenous AR-V7 (AR-V7) and EGFP-tagged AR-V7 (EGFP-AR-V7) in LNCaP (**C**) and 22Rv1 (**D**) cells. β-actin was used as an internal control.

To study the effects of AR-V7 on NF-κB, prostate cancer LNCaP and 22Rv1 cells were transfected with pEGFP, pEGFP-AR-FL, or pEGFP-AR-V7. As shown in Figure 2A,B, endogenous expression of full-length AR was detected in LNCaP and 22Rv1 cells using an AR antibody. Androgen receptor splice variants (AR-Vs) were also detected in 22Rv1, but not in LNCaP cells. The extra band in lane pEGFP-AR-FL and the extra band in lane pEGFP-AR-V7 detected in both LNCaP and 22Rv1 cells belong to the EGFP-tagged full-length AR and EGFP-tagged AR-V7, respectively (Figure 2A,B). When a specific antibody against AR-V7 was used, endogenous expression of AR-V7 was detected in 22Rv1 cells but not in LNCaP cells. The expression of EGFP-tagged AR-V7 was detected in LNCaP and 22Rv1 cells transfected with pEGFP-AR-V7 plasmid (Figure 2C,D).

### 2.3. Effects of AR-V7 on NF-κB Activity

**Figure 3:**
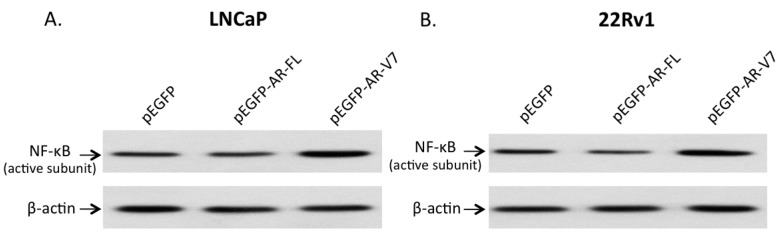
Expression of active subunit of nuclear factor-kappa B (NF-κB) in transfected prostate cancer cells. LNCaP (**A**) and 22Rv1 (**B**) cells were transfected with pEGFP, pEGFP-AR-FL, or pEGFP-AR-V7 expression plasmids. Immunoblot using an antibody against the active subunit of NF-κB was carried out on protein lysates from transfected cells. β-actin was used as an internal control.

**Figure 4:**
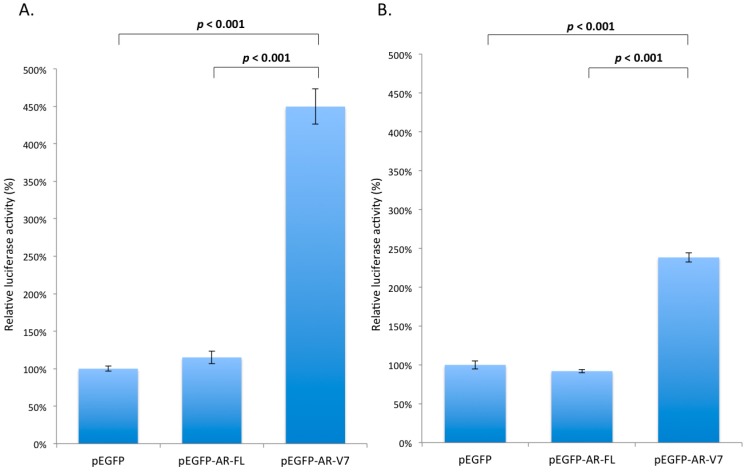
NF-κB reporter activities in transfected prostate cancer cells. LNCaP (**A**) and 22Rv1 (**B**) cells were transfected with pEGFP, pEGFP-AR-FL, or pEGFP-AR-V7 expression plasmids. Luciferase reporter assay was used to measure NF-κB activities in those transfected cells. Cells transfected with pEGFP were used as a control. Data are shown as relative luciferase activity (%) ± S.E.

**Figure 5:**
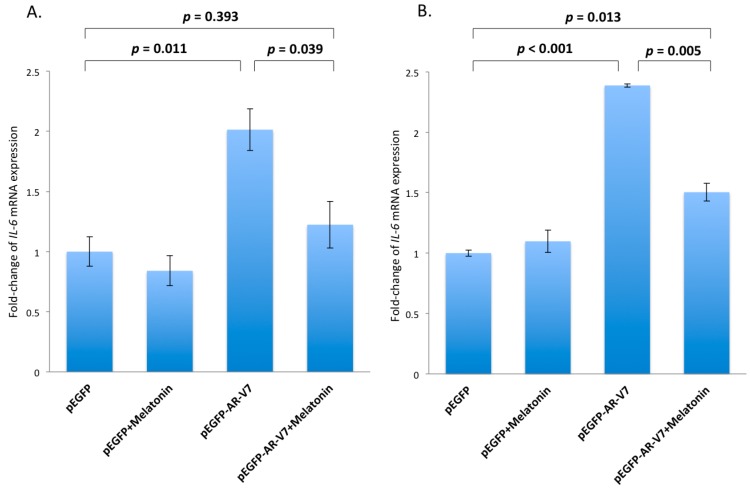
Q-PCR analysis of interleukin *(IL)-6* expression in transfected prostate cancer cells. LNCaP (**A**) and 22Rv1 (**B**) cells were transfected with pEGFP or pEGFP-AR-V7 expression plasmids, in the presence or absence of melatonin (10^−6^ M) for 24 h. The relative levels of *IL-6* were compared using cells transfected with pEGFP as a control. Data are shown as relative fold-change of *IL-6* mRNA expression ± S.E.

To investigate any functional interactions between AR-V7 and NF-κB, expression of the active form of NF-κB in LNCaP and 22Rv1 cells, transfected with pEGFP-AR-FL or pEGFP-AR-V7, was detected. As shown in Figure 3A,B, expression of the active form of NF-κB was elevated approximately 2.7-fold and 1.5-fold, by densitometry, in LNCaP and 22Rv1 cells overexpressing AR-V7, respectively, compared with the pEGFP- or pEGFP-AR-FL-transfected cells. To demonstrate functional activation of NF-κB by AR-V7, NF-κB activities in LNCaP and 22Rv1 cells transfected with plasmid pEGFP-AR-FL or pEGFP-AR-V7 were measured by luciferase reporter assays. As shown in Figure 4, the NF-κB reporter activities were elevated by 4.5-fold (*p* < 0.001) and 2.4-fold (*p* < 0.001), in LNCaP (Figure 4A) and 22Rv1 cells (Figure 4B) transfected with pEGFP-AR-V7, respectively, compared to the empty plasmid vector pEGFP-transfected cells. No up-regulation of NF-κB reporter activity was detected in LNCaP and 22Rv1 cells transfected with pEGFP or pEGFP-AR-FL plasmids (Figure 4). These results suggest that AR-V7 can activate NF-κB in prostate cancer cells. To further confirm NF-κB activation by AR-V7, the expression of *IL-6*, a well-known downstream target gene of the transcription factor NK-κB [28], was analyzed using quantitative polymerase chain reaction (Q-PCR). As shown in Figure 5, *IL-6* transcription was significantly up-regulated by 2-fold (*p* = 0.011) in LNCaP cells (Figure 5A), and by 2.4-fold (*p* < 0.001) in 22Rv1 cells (Figure 5B) overexpressing AR-V7, as compared to pEGFP-transfected cells.

### 2.4. Inhibition of AR-V7 Induced IL-6 Gene Expression by Melatonin

In light of our present results which showed that AR-V7 could activate NF-κB with resultant up-regulation of *IL-6* and our previous data which showed inhibition of activated NF-κB signaling by melatonin in prostate cancer cells [23], we proceeded to test whether or not melatonin could inhibit the AR-V7-induced *IL-6* gene expression in LNCaP and 22RV1 cells. In LNCaP cells, AR-V7 up-regulated the expression of *IL-6* by 2-fold (*p* = 0.011). However, the stimulatory effects of AR-V7 on *IL-6* expression could be significantly reduced by 10^−6^ M melatonin treatment (*p* = 0.039) (Figure 5A). It is noteworthy that in the presence of melatonin, AR-V7 could not up-regulate the expression of *IL-6* (*p* = 0.393), indicating that melatonin could abrogate the increase in *IL-6* gene expression induced by AR-V7 overexpression in LNCaP cells (Figure 5A). On the other hand, melatonin could significantly (*p* = 0.005) attenuate the AR-V7 induced 2.4-fold increase in *IL-6* expression (Figure 5B) in 22Rv1 cells transfected with pEGFP-AR-V7.

### 2.5. Inhibition of NF-κB Induced AR-V7 Expression by Melatonin

**Figure 6:**
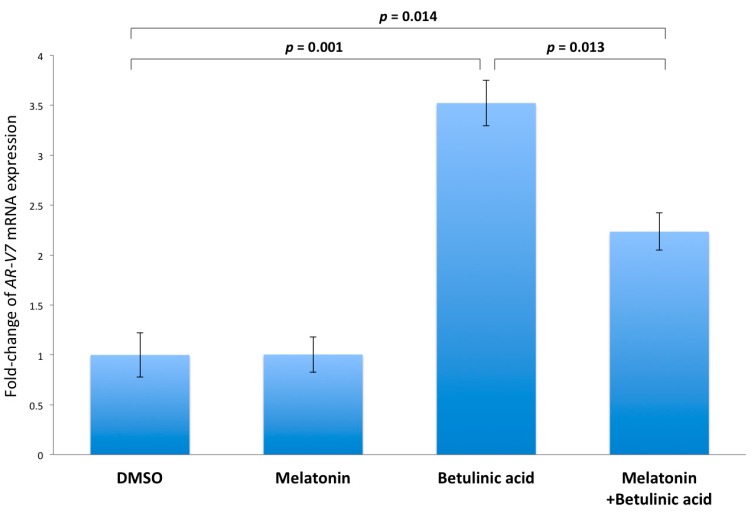
Q-PCR analysis of androgen receptor splice variant-7 (*AR-V7)* mRNA expression. LNCaP cells were treated with 10^−6^ M melatonin, 10^−6^ M betulinic acid, 10^−6^ M melatonin plus 10^−6^ M betulinic acid, or 0.001% dimethylsulfoxide (DMSO) for 48 h. The relative levels of *AR-V7* were then measured by Q-PCR and were compared to cells treated with DMSO as a control. Data are shown as relative fold-change of *AR-V7* mRNA expression ± S.E.

It has been reported that activation of NF-κB could induce *AR-V7* mRNA expression [29]. To confirm the above finding, betulinic acid, which is a NF-κB activator, was used to activate NF-κB, and the expression level of *AR-V7* was then measured by Q-PCR. To observe the induction of *AR-V7* by activated NF-κB, LNCaP but not 22Rv1 cells were used because 22Rv1 cells are already expressing highly elevated AR-V7 levels compared to LNCaP cells (Figure 2C,D). As shown in Figure 6, treatment of LNCaP cells with 10^−6^ M betulinic acid significantly (*p* = 0.001) elevated the expression of *AR-V7* by 3.5-fold, as compared to the DMSO-treated cells (control). Of note, the betulinic acid-stimulated expression of *AR-V7* could be significantly reduced (*p* = 0.013) by co-incubation of the LNCaP cells with 10^−6^ M melatonin.

### 2.6. Involvement of Membrane MT_1_ Receptor in Melatonin’s Inhibitory Effect on AR-V7-Induced NF-κB Activation

**Figure 7:**
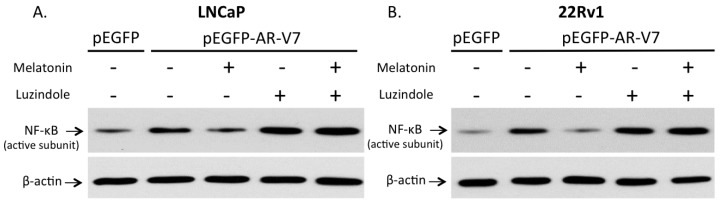
Effects of melatonin with or without luzindole on the expression of active subunit of NF-κB in transfected prostate cancer cells. LNCaP (**A**) and 22Rv1 (**B**) cells were transfected with pEGFP, or pEGFP-AR-V7, in the presence or absence of 0.001% dimethylsulfoxide (DMSO), 10^−6^ M melatonin, 10^−6^ M luzindole, or 10^−6^ M melatonin plus 10^−6^ M luzindole. Immunoblot using an antibody against the active subunit of NF-κB was carried out on protein lysates from transfected cells. β-actin was used as an internal control.

While it has been recently reported by us that melatonin can inhibit the constitutively active NF-κB via membrane G protein-coupled MT_1_ receptor in LNCaP and 22Rv1 prostate cancer cells [23], we would like to confirm that the MT_1_ receptor is responsible for mediating the inhibitory effect of melatonin on AR-V7 induced NF-κB activation observed in the present investigation. LNCaP and 22Rv1 cells were transfected with pEGFP or pEGFP-AR-V7 expression plasmid. The pEGFP-AR-V7-transfected cells were treated separately with 10^−6^ M melatonin, 10^−6^ M luzindole (a melatonin receptor antagonist), or 10^−6^ M melatonin plus 10^−6^ M luzindole. After 48 h of treatment with melatonin with or without luzindole, NF-κB activation in transfected cells was detected by immunoblot. As shown in Figure 7, expression of active NF-κB was increased after LNCaP and 22Rv1 cells were transfected with pEGFP-AR-V7. Of note, the activating effects of AR-V7 on NF-κB were blocked by 10^−6^ M melatonin. Furthermore, the inhibitory effects of melatonin on AR-V7-mediated NF-κB activation could be blocked by 10^−6^ M luzindole. In separate experiments, LNCaP and 22Rv1 cells were transfected with pEGFP expression plasmid and after 48 h of treatment with or without luzindole, NF-κB activation in transfected cells was detected by immunoblot. As shown in Figure S1, no effects of luzindole on active NF-κB levels in LNCaP and 22Rv1 cells overexpressing the pEGFP vector were observed. These results confirm that the observed inhibition of AR-V7 induced NF-κB activation by melatonin in the present investigation is mediated via membrane MT_1_ receptor, which has been shown by us previously to play an important role in transducing the antiproliferative action of melatonin on prostate cancer cells [23].

## 3. Discussion

Prostate cancer has become a major health problem of elderly men in the western world. Men with advanced or metastatic disease receive standard palliative castration or ADT [2]. Sadly, these patients inevitably succumb to their relapsed tumors after a variable period of disease remission. Recently approved drugs, abiraterone and enzalutamide, which further enhance androgen depletion by targeting the androgen receptor signaling pathway, are a welcomed breakthrough in the treatment of CRPC [3,4,5,6]. Like other effective anti-cancer drugs, the development of tumor resistance toward abiraterone or enzalutamide is a major concern and challenge to scientists and clinicians. Together, these create the demand for novel therapeutic strategies against advanced or metastatic prostate cancer as well as abiraterone-resistant or enzalutamide-resistant CRPC.

It is worth noting that melatonin, which acts by inhibiting both activated AR and NF-κB signaling in prostate cancer cells, has been demonstrated to be a novel prostate growth inhibitor [22,23]. Given that constitutively active androgen receptor splice variants, in particular AR-V7, have been shown to confer resistance to abiraterone or enzalutamide [13,14], and that activation of NF-κB [24,25] and reactivation of AR signaling [26,27] are involved in prostate cancer progression and CRPC development, it would be of interest to investigate any interactions among AR-V7, NF-κB, and melatonin in prostate cancer cells to gain further insights on the therapeutic potential of melatonin in advanced prostate cancer and CRPC management.

In this study, we demonstrated that transient ectopic AR-V7 overexpression in LNCaP and 22Rv1 prostate cancer cells activate NF-κB with a resultant increase in *IL-6* gene expression (Figure 3, Figure 4 and Figure 5). LNCaP and 22Rv1 cells were used in the present investigation because these two prostate cancer cell lines have been previously shown by us to be responsive to melatonin receptor-mediated antiproliferative actions [20,21,22,23]. Interestingly, melatonin, which has been shown by us to inhibit constitutively active NF-κB in prostate cancer cells via the membrane G protein-coupled MT_1_ receptor [23], reduces both AR-V7-induced NF-κB activation and *IL-6* gene transcription in LNCaP and 22Rv1 cells (Figure 5 and Figure 7). Given that IL-6 is an autocrine and paracrine growth factor involved in CRPC development, prostate cancer metastasis and chemo-resistance of prostate cancer [28,30,31,32,33], our results suggest that the effects of AR-V7 on CRPC development and prostate cancer progression may be mediated in part via activation of NF-κB/IL-6 signaling in the tumor cells. Intrigued by recent reports on increased AR-V7 expression in prostate cancer and epithelial cells as a result of NF-κB activation [29,34], we have also tested whether melatonin can inhibit *AR-V7* mRNA expression induced by activation of NF-κB signaling in LNCaP cells. Importantly, treatment of LNCaP cells with melatonin attenuates the betulinic acid-induced rise in *AR-V7* mRNA expression (Figure 6). Taken together, the data support the presence of bi-directional positive interactions between AR-V7 expression and NF-κB signaling in CRPC pathogenesis and suggest that melatonin may be of promise as a potential therapeutic agent against AR-V7-mediated CRPC by serving as a NF-κB inhibitor. It is worth noting that we have also attempted to study chronic AR-V7 overexpression in LNCaP cells, which express a low endogenous *AR-V7* transcript level [35]. Disappointingly, selected LNCaP cell clones which had been stably-transfected with pEGFP-AR-V7 expression vector failed to grow under our experimental conditions.

**Figure 8:**
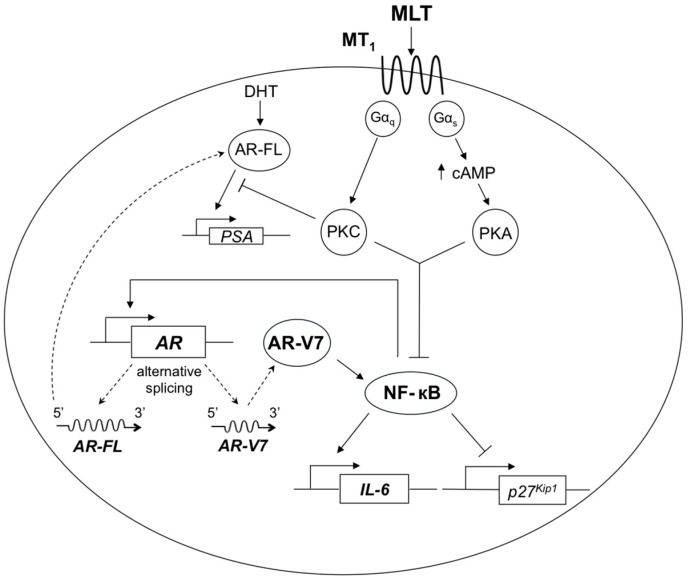
Schematic diagram showing the signaling pathways involved in melatonin receptor-mediated growth inhibition of prostate cancer cells. → denotes activation (may involve multi-steps); ⊣ denotes inhibition (may involve multi-steps). AR-FL and AR-V7 are full-length and truncated forms of AR, respectively, generated by alternative splicing of AR precursor mRNAs. Dotted line denotes transcription or translation. Abbreviations: MLT, melatonin; PKC, protein kinase C; PKA, protein kinase A; NF-κB, nuclear factor-kappa B; DHT, dihydrotestosterone; AR, androgen receptor; AR-FL, full-length androgen receptor; AR-V7, androgen receptor splice variant-7; IL-6, interleukin-6; PSA, prostate-specific antigen.

Based on the present results, we have updated our previously-reported melatonin MT_1_ receptor antiproliferative pathway in prostate cancer [23] with bi-directional positive interactions between AR-V7 and NF-κB/IL-6 signaling (Figure 8). Melatonin, by inhibiting NF-κB activation via the previously-reported MT_1_ receptor-mediated antiproliferative pathway, can disrupt bi-directional positive interactions between AR-V7 and NF-κB signaling, thereby possibly delaying the development of castration resistance in advanced prostate cancer. Thus, melatonin, when used in combination treatment with ADT, may provide additional therapeutic benefits in patients with advanced prostate cancer or CRPC. Moreover, besides being a small molecule with an excellent pharmacological safety profile, melatonin has been shown by a recent systematic review and meta-analysis of randomized trials to significantly reduce asthenia, leucopenia, nausea and vomiting, hypotension, and thrombocytopenia in cancer patients who are also receiving chemotherapy, radiotherapy, supportive therapy, or palliative therapy [36]. We believe that this novel approach of combined androgen depletion and melatonin repletion may improve both the survival and quality of life of patients suffering from advanced prostate cancer or CRPC. Apparently, this therapeutic potential of melatonin, via combined androgen depletion and melatonin repletion, is worth translation in the clinic for advanced prostate cancer/CRPC management.

## 4. Materials and Methods

### 4.1. Cells, Plasmids, and Chemicals

Human prostate cancer cell lines LNCaP.FGC (CRL-1740) and 22Rv1 (CRL-2505) were purchased from American Type Culture Collection (Manassas, VA, USA). LNCaP and 22Rv1 cells were grown and maintained in Roswell Park Memorial Institute (RPMI) 1640 medium without phenol red (Life Technologies, Inc., Grand Island, NY, USA) supplemented with l-glutamine and 10% fetal bovine serum (FBS) (Life Technologies). To minimize the influence of hormones present in normal FBS on our experiments, charcoal stripped FBS (Cat. No. F6765, Sigma-Aldrich, St. Louis, MO, USA) was used in all experiments involving 22Rv1. However, normal FBS was used in LNCaP cells whose growth was significantly compromised in charcoal stripped FBS. The expression plasmids which contain full-length AR (pEGFP-AR-FL) and AR-V7 (pEGFP-AR-V7) were kindly provided by Dr. J. Luo (Johns Hopkins University, Baltimore, MD, USA). The cDNAs encoding AR-V7 and full-length AR were inserted into the pEGFP-C3 vector to express the EGFP-AR-V7 and EGFP-AR-FL fusion protein respectively [8,37]. Melatonin (Cat. No. M5250) and luzindole (Cat. No. L2407) were purchased from Sigma-Aldrich. Betulinic acid, which is a NF-κB activator, was obtained from Tocris (Bristol, UK).

### 4.2. Fluorescence Microscopy

LNCaP and 22Rv1 cells were grown in 12-well plates until 50–60% confluency. The cells were then transfected with 0.5 µg of empty pEGFP vector, 0.5 µg of pEGFP-AR-FL, or 0.5 µg of pEGFP-AR-V7, using FuGENE^®^ HD Transfection Reagent (Cat. No. E231A; Promega, Madison, WI, USA). After 24 h of incubation, any fluorescence signals from the recombinant EGFP or EGFP-tagged proteins in the transfected cells were observed under a fluorescence microscope.

### 4.3. Immunoblot Analysis

LNCaP and 22Rv1 cells were transfected with pEGFP, pEGFP-AR-FL, or pEGFP-AR-V7 for 24 h. The cells were then lysed in SDS sample buffer, and the proteins were harvested for immunoblotting as described previously [21]. Expression of AR and its variants were detected by a primary antibody against AR (sc-7305, 1:2000 dilution; Santa Cruz Biotechnology, Santa Cruz, CA, USA) and a secondary antibody against mouse immunoglobulin G (Cell Signaling Technology, Danvers, MA, USA). A specific primary antibody against AR-V7 (AG10008, 1:2000 dilution; Precision Antibody, Columbia, MD, USA) and a secondary antibody against mouse immunoglobulin G (Cell Signaling Technology) were also used to detect the expression of AR-V7 in the transfected prostate cancer cells. In addition, the detection of NF-κB activation in pEGFP-, pEGFP-AR-FL-, and pEGFP-AR-V7-transfected LNCaP and 22Rv1 cells were performed using a primary antibody against the active subunit of NF-κB (MAB3026, 1:2000 dilution; Millipore, CA, USA) and a secondary antibody against mouse immunoglobulin G (Cell Signaling Technology). In separate sets of experiments, LNCaP and 22Rv1 cells were transfected with pEGFP or pEGFP-AR-V7 in the presence or absence of 0.001% dimethylsulfoxide (DMSO), 10^−6^ M melatonin, 10^−6^ M luzindole, or 10^−6^ M melatonin plus 10^−6^ M luzindole. After 48 h, activation of NF-κB was detected using a primary antibody against the active subunit of NF-κB (Millipore) and a secondary antibody against mouse immunoglobulin G (Cell Signaling Technology). The signals were visualized by enhanced chemiluminescence Western blotting system (Bio-Rad, Hercules, CA, USA). All blots were stripped in 25 mM glycine buffer (pH 2.0) for 30 min before re-probing with β-actin (sc-5286, 1:5000 dilution; Santa Cruz Biotechnology).

### 4.4. Luciferase Reporter Assays

LNCaP and 22Rv1 cells (density 5 × 10^4^/mL) were seeded in 24-well plates (*n* = 6). After 24 h, the cells in each well were transfected with a mixture of 1.0 µL FuGENE^®^ HD Transfection Reagent (Promega, Madison, WI, USA), 0.2 µg NF-κB reporter plasmid pNF-κB-TA-Luc (Takara Bio Inc., Shiga, Japan), 0.04 µg *Renilla* luciferase reporter pRL-tk (Promega), and 0.1 µg pEGFP, 0.1 µg pEGFP-AR-FL, or 0.1 µg pEGFP-AR-V7. The NF-κB reporter activities were measured 24 h after transfection using the Dual-Luciferase^®^ Reporter Assay System (Promega). Both the firefly and Renilla luciferase activities were measured according to the manufacturer’s instructions. The cells were washed three times with phosphate-buffered saline. Passive lysis buffer was added to each well and was shaken at room temperature for 15 min. Lysate (20 µL) from each sample was transferred to 96-well plate for firefly and Renilla luciferase activity measurements by a luminometer [20].

### 4.5. Quantitative Polymerase Chain Reaction (Q-PCR)

LNCaP and 22Rv1 cells were seeded in 6-well plates. At 50–60% confluency, the cells were transfected with 0.2 µg pEGFP or 0.2 µg pEGFP-AR-V7. After 24 h of transfection, the cells were treated with 10^−6^ M melatonin or 0.001% DMSO as a control. Total RNA was extracted from the cells and reversely transcribed into cDNAs after a further 24 h of incubation. The expression of *IL-6* was measured by Q-PCR, using a specific forward primer 5′-ATGCAATAACCACCCCTGAC-3′ and a specific reverse primer 5′-GAGGTGCCCATGCTACATTT-3′. In separate sets of experiments, LNCaP cells reaching 50–60% confluency in 6-well plates were incubated with 10^−6^ M melatonin, 10^−6^ M betulinic acid, 10^−6^ M melatonin plus 10^−6^ M betulinic acid, or 0.001% dimethylsulfoxide (DMSO) for 48 h, before the levels of endogenous *AR-V7* were measured by Q-PCR, using a specific forward primer 5′-CCATCTTGTCGTCTTCGGAAATGTTATGAAGC-3′ and a specific reverse primer 5′-TTTGAATGAGGCAAGTCAGCCTTTCT-3′. Briefly, 4 µL of diluted cDNAs, 1 µL of forward/reverse primers, and 5 µL of 2× iTaq™ Universal SYBR^®^ Green Supermix (Bio-Rad) were added to form a 10 µL PCR reaction mixture. Q-PCR was performed using MyiQ (Bio-Rad). The conditions were 95 °C for 5 min, followed by 40 cycles at 95 °C for 15 s and 60 °C for 35 s. Relative quantities of specific RNAs were calculated using comparative CT method. The fold difference was normalized to endogenous β-actin, which was detected by Q-PCR using a specific forward primer 5′-GGACTTCGAGCAAGAGATGG-3′ and a specific reverse primer 5′-AGCACTGTGTTGGCGTACAG-3′.

### 4.6. Statistical and Data Analyses

The data are presented as mean ± S.E. and analyzed by unpaired Student’s *t* test. The level of significance for all statistical analyses was set at *p* < 0.05*.*

## 5. Conclusions

In this study, we demonstrated that melatonin inhibits AR-V7-induced NF-κB activation and NF-κB activator-induced *AR-V7* expression in prostate cancer cells. The data support the potential use of melatonin in CRPC therapy.

## Supplementary Materials

Supplementary materials can be found at www.mdpi.com/1422-0067/18/6/1130/s1.
