# Supplementary figures and images for: Melatonin Inhibits Androgen Receptor Splice Variant-7 (AR-V7)-Induced Nuclear Factor-Kappa B (NF-κB) Activation and NF-κB Activator-Induced AR-V7 Expression in Prostate Cancer Cells: Potential Implications for the Use of Melatonin in Castration-Resistant Prostate Cancer (CRPC) Therapy
Source: Int J Mol Sci. 2017 May 31;18(6):1130. doi: 10.3390/ijms18061130 (PMC5485954; doi:10.3390/ijms18061130)

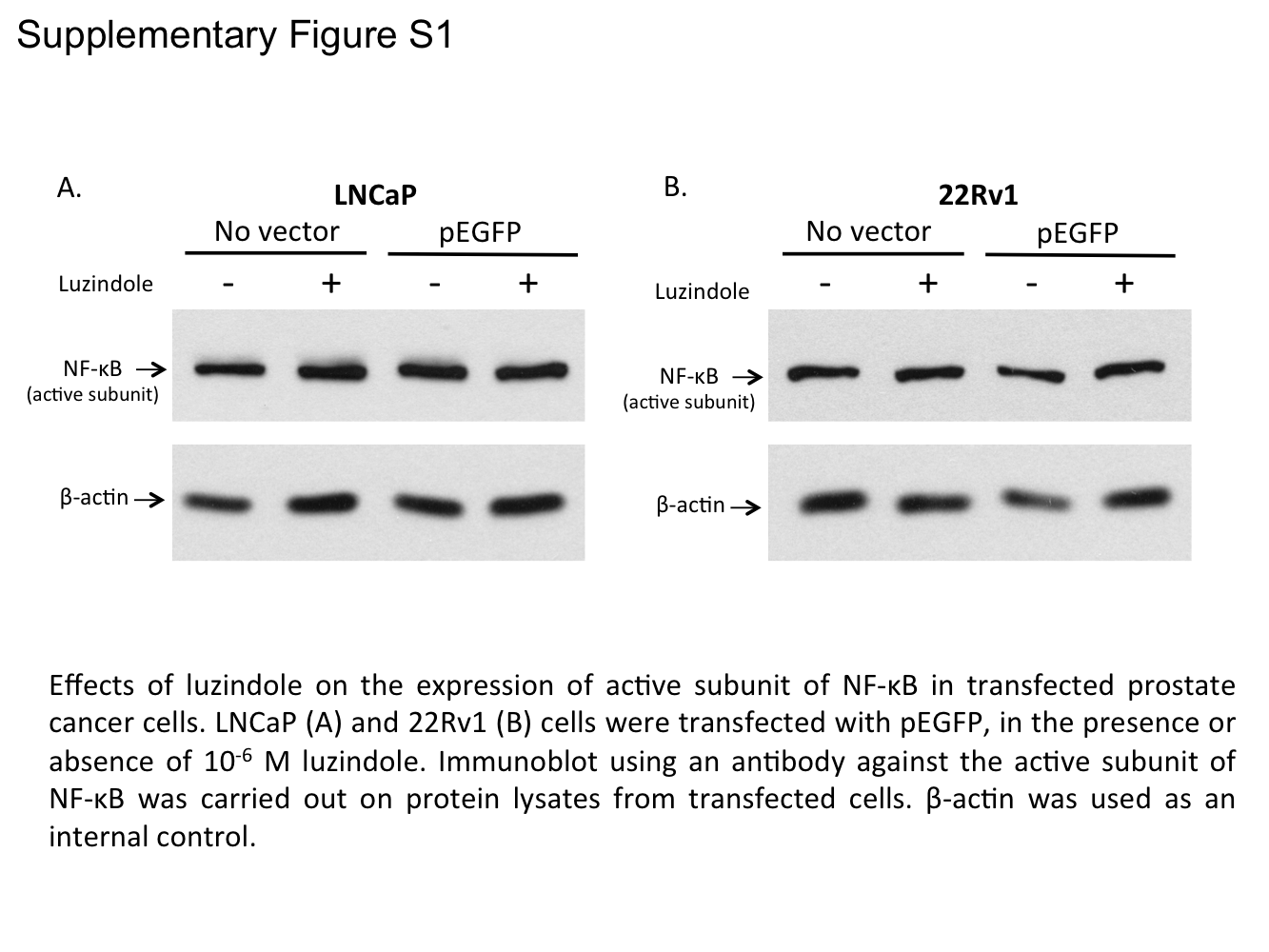

Supplement: Supplementary file 1 [file ijms-18-01130-s001.tif]
